# Simple risk factors to predict urgent endoscopy in nonvariceal upper gastrointestinal bleeding pre-endoscopically

**DOI:** 10.1097/MD.0000000000003603

**Published:** 2016-07-01

**Authors:** Jianzong Wang, Duanming Hu, Wen Tang, Chuanyin Hu, Qin Lu, Juan Li, Jianhong Zhu, Liming Xu, Zhenyu Sui, Mingjie Qian, Shaofeng Wang, Guojian Yin

**Affiliations:** Department of Gastroenterology, the Second Affiliated Hospital of Soochow University, Suzhou, Jiangsu Province, People's Republic of China.

**Keywords:** nonvariceal upper gastrointestinal bleeding, risk score, urgent endoscopy

## Abstract

The goal of this study is to evaluate how to predict high-risk nonvariceal upper gastrointestinal bleeding (NVUGIB) pre-endoscopically. A total of 569 NVUGIB patients between Match 2011 and January 2015 were retrospectively studied. The clinical characteristics and laboratory data were statistically analyzed. The severity of NVUGIB was based on high-risk NVUGIB (Forrest I–IIb), and low-risk NVUGIB (Forrest IIc and III). By logistic regression and receiver-operating characteristic curve, simple risk score systems were derived which predicted patients’ risks of potentially needing endoscopic intervention to control bleeding. Risk score systems combined of patients’ serum hemoglobin (Hb) ≤75 g/L, red hematemesis, red stool, shock, and blood urine nitrogen ≥8.5 mmol/L within 24 hours after admission were derived. As for each one of these clinical signs, the relatively high specificity was 97.9% for shock, 96.4% for red stool, 85.5% for red hematemesis, 76.7% for Hb ≤75 g/L, and the sensitivity was 50.8% for red hematemesis, 47.5% for Hb ≤75 g/L, 14.2% for red stool, and 10.9% for shock. When these 5 clinical signs were presented as a risk score system, the highest area of receiver-operating characteristic curve was 0.746, with sensitivity 0.675 and specificity 0.733, which discriminated well with high-risk NVUGIB. These simple risk factors identified patients with high-risk NVUGIB of needing treatment to manage their bleeding pre-endoscopically. Further validation in the clinic was required.

## 1 Introduction

Nonvariceal upper gastrointestinal bleeding (NVUGIB) is a common medical emergency. The incidence of NVUGIB has been reported to range from 50 to 150 per 100,000 adults/y,^[1,2]^ and mortality rates range between 8% and 14%.^[2]^

In the guideline for diagnosis and management of NVUGIB patients, emergent endoscopic examination and risk stratification, which play an important role in the diagnosis, are recommended in the first 24 to 48 hours,^[3–5]^ as soon as possible. Combination therapy of medicine and endoscopic hemostasis is recommended as the first choice of treatment of NVUGIB.^[6]^

However, although it is strongly suggested that endoscopic examination should be completed as soon as possible in those patients with NVUGIB, the time of emergency endoscopy cannot be completely unified due to the different operation modes and medical conditions. And another unresolved problem is whether all those NVUGIB patients do really need emergent endoscopic examination and endoscopic hemostasis in the first 24 to 48 hours? Do all those NVUGIB patients really need the endoscopic hemostasis, like submucosal injection of epinephrine, electric coagulation, titanium clip, and argon knife as soon as possible? In addition, the condition of those patients is always not very well for urgent endoscopic examination and hemostasis. The stomach might be full of blood material or foodstuff during urgent endoscopy, which makes endoscopic examination with a very bad vision. Also the risks of aspiration pneumonia and aspiration asphyxia are increased during endoscopic examination when the NVUGIB patients were with active bleeding. Moreover, unstable vital signs could bring a lot of unexpected medical accidents during endoscopy.

In fact, low-risk NVUGIB patients with emergency endoscopy do make limited emergency resources or green channel less and less efficient.

Can we get a risk scoring system, which could help us to differentiate those high-risk NVUGIB patients who should be done previous to endoscopy and who do not need? Giese et al^[7]^ supposed that no relevant pre-endoscopic variables for the prediction of active UGIB at emergency endoscopy could be found, and that pre-endoscopic evaluation cannot replace rapid endoscopy. In this study, our goal was to evaluate how to predict high-risk NVUGIB patients for urgent endoscopy. It was showed that a simple risk score could potentially identify patients at low or high risk of needing emergent management of their bleeding pre-endoscopically.

## 2 Materials and methods

The Medical Ethics Committee of a 3-A hospital, the 2nd Affiliated Hospital of Suzhou University, Suzhou, China, approved the study. Due to the retrospective nature of the study, informed consent was waived. NVUGIB was diagnosed according to the clinical presentations and endoscopic findings.^[8]^ Inclusion criteria were as follows: patients diagnosed as having UGIB, presented with hematemesis and (or) melena, and without esophageal and (or) gastric varices confirmed by endoscopy for those patients within 48 hours after the onset of the clinical presentation.

Between Match 2011 and January 2015, a total of 569 NVUGIB patients were retrospectively studied, including 484 (85.06%) with peptic ulcers, 26 (4.57%) with gastric cancers, 17 (2.99%) with dieulafoy, 12 (2.11%) with acute hemorrhagic gastritis, and 30 (5.27%) with Mallory–Weiss syndromes. Medical history was carefully recorded, especially the history of nonsteroidal anti-inflammatory drug (NSAID) consumption, liver cirrhosis, hypertension, diabetes mellitus, cardiovascular diseases, peptic ulcer, and weight loss. The clinical presentations were also recorded, including palpitation, cold sweat, syncope, systolic blood pressure (SBP), diastolic blood pressure (DBP), heart rates, shock state, color and volume of vomited material, and color and volume of blood stool. All patients were treated with comprehensive routine therapy according to the guidelines for diagnosis and treatment of NVUGIB.^[9–11]^

Laboratory data of all these NVUGIB patients within 48 hours after the onset of classical hematemesis and (or) melena were also chosen to do the statistical analysis. The lowest levels of hemoglobin (Hb), erythrocyte mean corpuscular volume (MCV), platelet cell (PLT), albumin, and the highest levels of prothrombin time (PT), activated partial thromboplastin time (APTT), blood urine nitrogen (BUN), and creatinine were reviewed.

The severity of NVUGIB was based on the Forrest classification, which was defined at endoscopy after 3 endoscopy specialists evaluated endoscopic presentations independently. Given in the Asia-Pacific Working Group consensus in 2011, an adherent clot on a peptic ulcer should be treated with endoscopy combined with a PPI if the clot cannot be removed,^[4]^ Forrest I to IIb was defined as high-risk NVUGIB, and Forrest IIc and III as low-risk NVUGIB in our study.^[12–14]^

Statistical evaluations were carried out using the SPSS (Statistical Package for Social Sciences) 13.0 software package. Numerical data were expressed as means ± standard deviation (SD) and categorical variables as means (ratio). Comparisons between high-risk NVUGIB and low-risk NVUGIB were performed by means of the 1-way analysis of variance (ANOVA) test for continuous variables, followed by Tukey honestly significant difference test when appropriate. Categorical variables were evaluated using Pearson chi-square test and Fisher exact test. Regression analysis was used for risk factors in continuous variables and categorical variables. Risk factors and different risk scores were evaluated by means of a receiver-operating characteristic (ROC) curve for selected cutoff points. Values of *P* < 0.05 were considered statistically significant.

## 3 Results

Total 569 NVUGIB patients, aged 53.02 ± 17.54 years (ranged 13–89 years), including 452 men (51.80 ± 18.10 years; range 13–89 years) and 117 women (57.15 ± 16.69 years; range 19–87 years) were retrospectively evaluated in this study. High-risk NVUGIB was diagnosed in 184 (32.34%) cases, and low-risk NVUGIB in 385 (67.66%) cases. Detailed results of the Forrest classification in the NVUGIB patients were as follows: Forrest Ia 9 (1.58%), Ib 41 (7.21%), IIa 74 (13.01%), IIb 59 (10.37%), IIc 52 (9.14%), and III 334 (58.70%). Between high-risk NVUGIB and low-risk NVUGIB, there were significant statistical differences in SBP, DBP, heart beats, Hb, HCT, PLT, albumin, BUN, volume and color of vomited material (red hematemesis), color of blood stool (red stool), palpitation, cold sweat, and shock (*P* < 0.05). Four (0.7%) patients died totally, in which 3 was in high-risk NVUGIB group, and 1 was in low-risk NVUGIB group. The clinical and laboratory data are shown in Table 1.

**Table 1:**
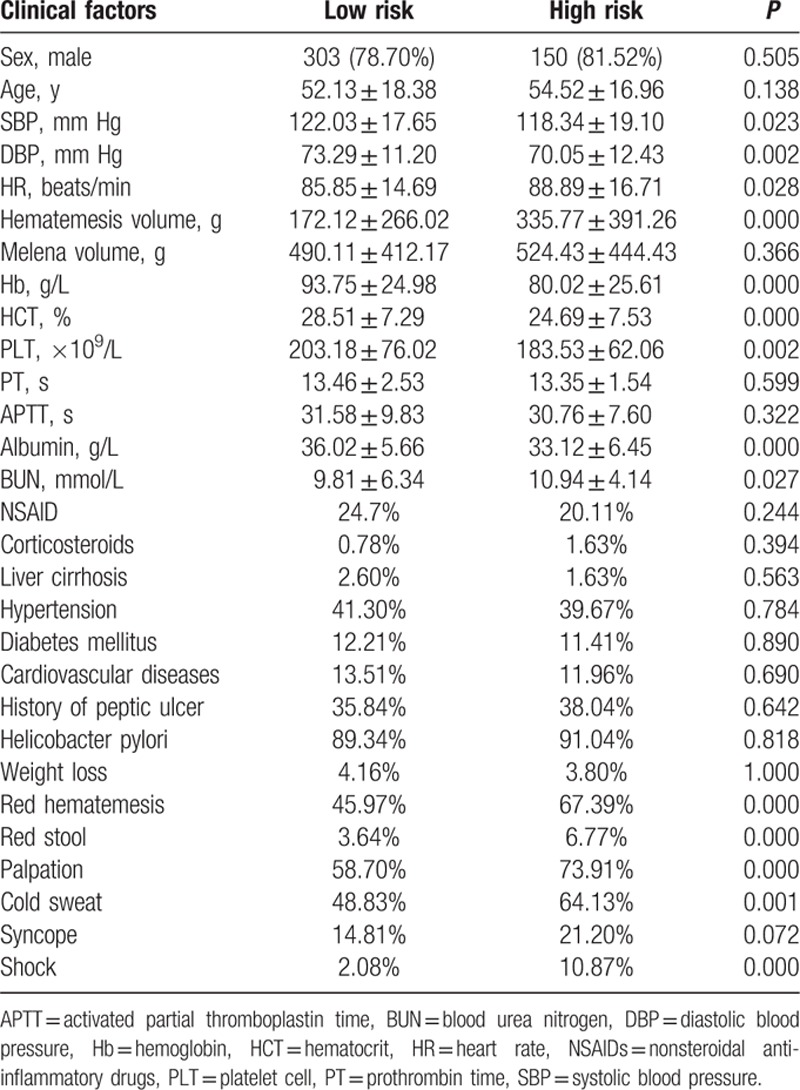
Clinical characteristics of 569 NVUGIB patients.

All the recorded categorical variables were used to do logistic regression analysis regarding clinical risk factors of high-risk NVUGIB. The color of hematemesis and melena was scored 1 when it was red hematemesis and stool (dark red, bright red, or containing red clot), and scored 0 when presented as other presentations. The risk was calculated as follows: 0.84 × red hematemesis + 1.42 × red stool + 1.419 × shock − 1.681. The Cox and Snell *R*^2^ and Nagelkerke *R*^2^ were 0.154 and 0.215, respectively, and the Exp (B) of red hematemesis, red stool, and shock was 2.316, 4.136, and 4.134, respectively (*P* < 0.01) (detailed in Table 2). When the continuous variables were used to do logistic regression analysis regarding clinical risk factors of high-risk NVUGIB, the risk was calculated as follows: 0.001 × hematemesis volume − 0.051 × Hb − 0.003 × PLT − 0.127 × PT + 0.038 × BUN + 2.25. The Cox and Snell *R*^2^ and Nagelkerke *R*^2^ were 0.154 and 0.215, respectively, and the Exp (B) of hematemesis, Hb, PLT, PT, and BUN was 1.001, 0.951, 0.997, 0.881, and 1.039 (*P* < 0.05) (Table 2).

**Table 2:**
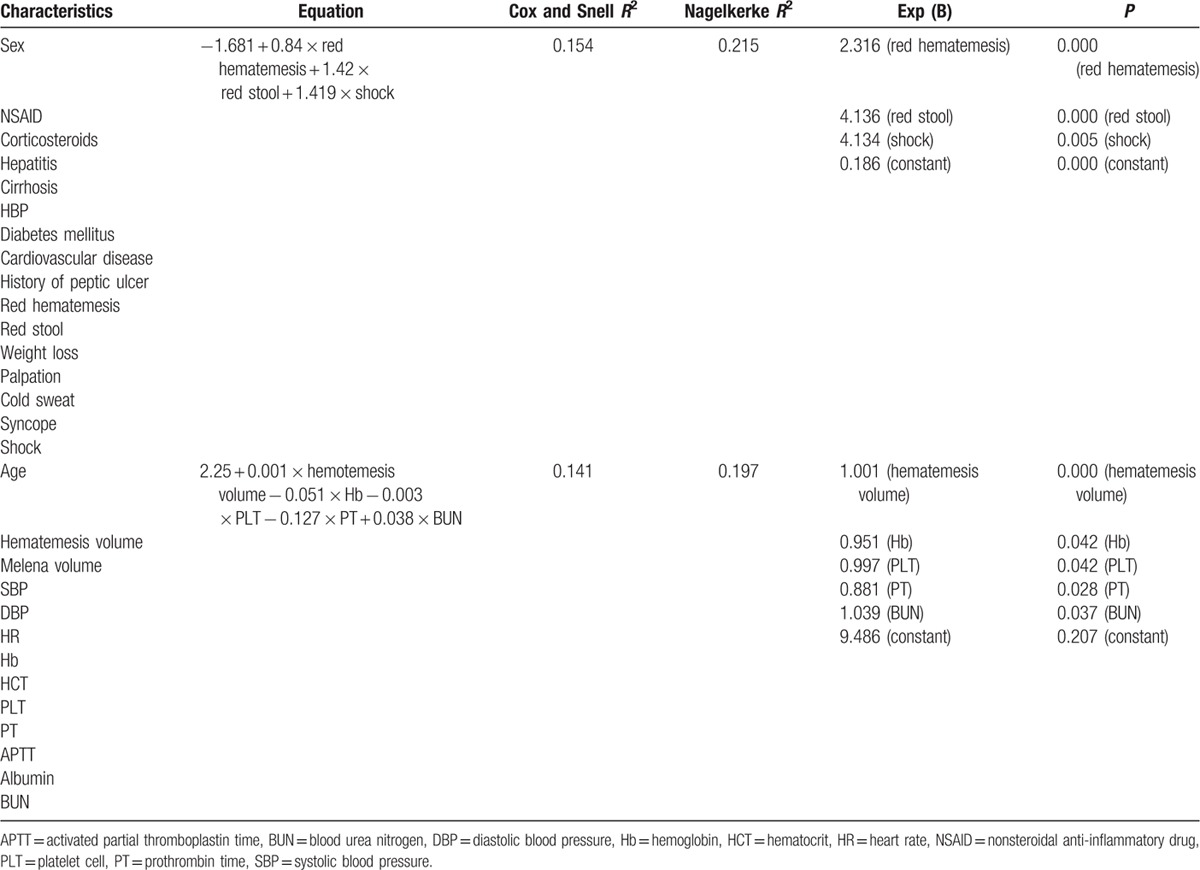
Logistic analysis of clinical and laboratory characteristics for high-risk NVUGIB patients.

**Figure 1:**
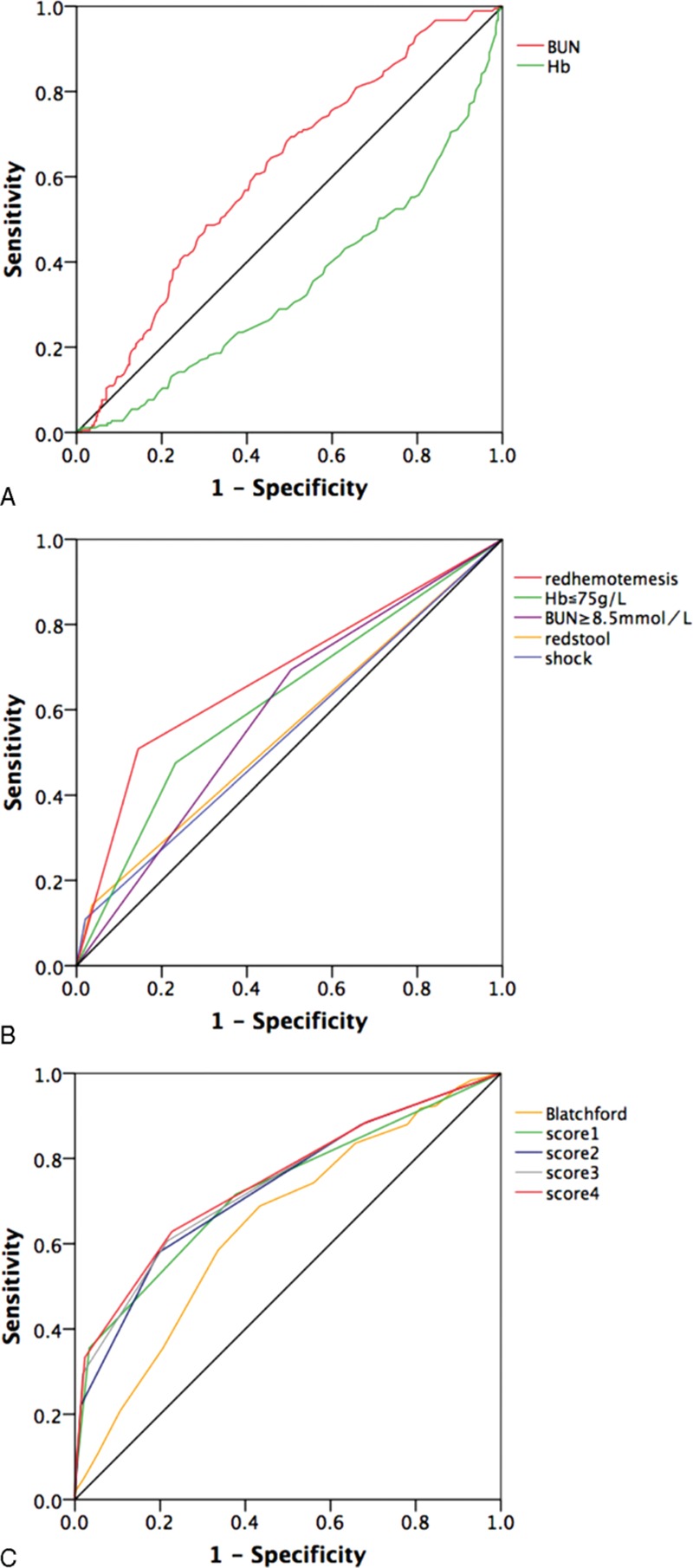
ROC analysis of risk factors in predicting high-risk NVUGIB. ROC = receiver-operating characteristic, NVUGIB = nonvariceal upper gastrointestinal bleeding.

Red hematemesis, Hb, shock, red stool, and BUN were chosen to do the further ROC analysis. The ROC method was used to distinguish high-risk NVUGIB from low-risk NVUGIB. When the Hb levels were evaluated first by means of ROC analysis, it was showed that when the cutoff point of Hb was 74.5 g/L, the area under the ROC curve (AUC), sensitivity, specificity, positive likelihood ratio (PLR), negative likelihood ratio (NLR), and Youden index were 0.348, 0.530, 0.227, 0.686, 0.483, and −0.243, respectively (*P* < 0.01). When the serum BUN levels were evaluated by means of ROC method, it was showed that when the cutoff point of BUN was 8.45 mmol/L, the AUC, sensitivity, specificity, PLR, NLR, and Youden index were 0.614, 0.694, 0.496, 1.737, 0.617, and 0.190, respectively (*P* < 0.01) (Table 3 and Fig. 1A). The red hematemesis, red stool, and shock have also been analyzed by ROC method, detailed in Table 3 and Fig. 1B. So Hb (≤75 g/L) and BUN (≥8.5 mmol/L) were selected to continue to assess new risk score systems regarding high-risk NVUGIB.

**Table 3:**
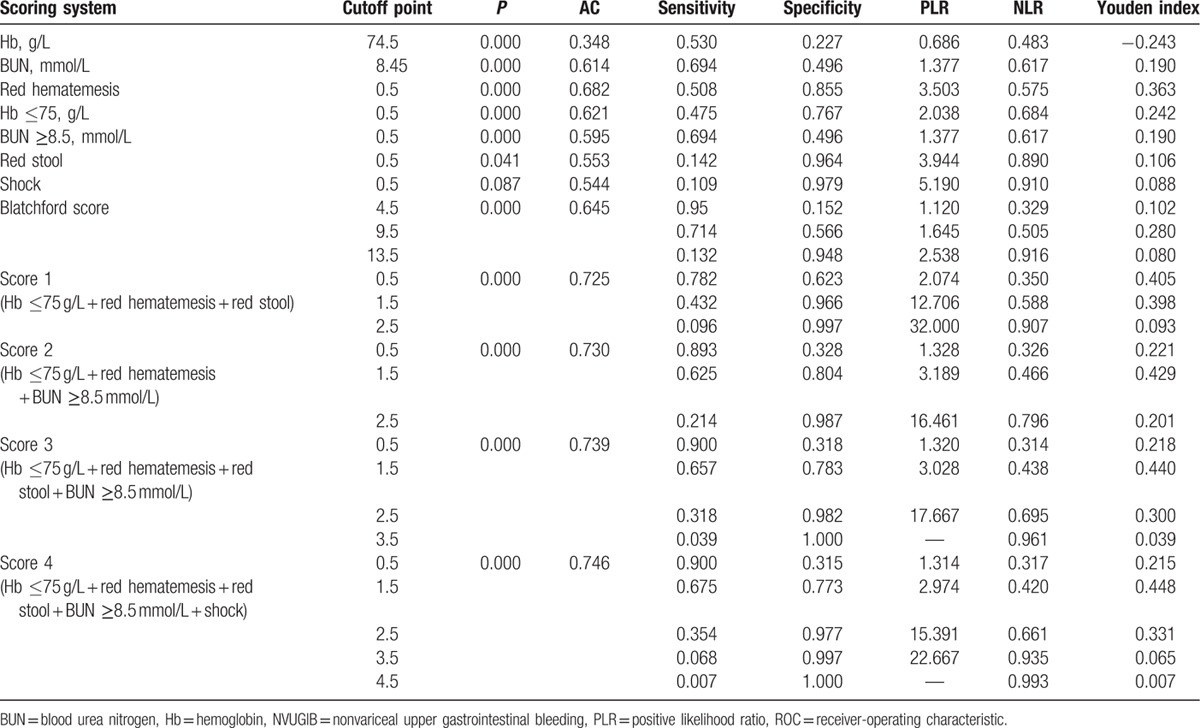
ROC analysis of clinical and laboratory characteristics for high-risk NVUGIB patients.

Different combinations of red hematemesis, Hb ≤75 g/L, and BUN ≥8.5 mmol/L, shock, red stool (each character was scored as 1) as different new score systems were used to do the further ROC analysis. New score systems discriminated well with high-risk NVUGIB (Table 3 and Fig. 1C). The highest area of ROC curves was 0.746, with sensitivity 0.675, specificity 0.733 in score 4 when the cutoff point was 1.5. Different cutoff points, with different *P* values, AUC, sensitivity, specificity, PLR, and NLR, are detailed in Table 3, with statistical significance (*P* < 0.01). Blatchford score was also compared with this new score system (Table 3). The ROC curves in the diagnosis of high-risk NVUGIB are presented in Fig. 1C.

## 4 Discussion

Urgent endoscopy in UGIB is an essential part of a complex medical care, which is highly reliable in identifying the cause and site of bleeding, and enables to start endoscopic hemostasis immediately and help to consider the prognosis of a patient.^[15]^ Mortality and probability of rebleeding has been reported to be related to the Forrest classification under emergent endoscopy, which was suggested to be the most useful risk score system for the prediction of rebleeding and death in patients with NVUGIB.^[8]^ In this study, Forrest classification was chosen to be the gold standard to define high-risk NVUGIB (Forrest I–IIb) and low-risk NVUGIB (Forrest IIb and III).^[13]^ It was found that high-risk NVUGIB was diagnosed in 184 (32.34%) cases, and low-risk NVUGIB was diagnosed in 385 (67.66%) cases, which was a little bit different from what Li^[12]^ reports in 2014. Li found that 437 (43.4%) were categorized with low-risk peptic ulcer bleeding (Forrest I–IIb) in a multicenter endoscopic survey of 1006 patients.

It was reported by Gisbert et al^[16]^ that high-risk variables of UGIB in a multivariate analysis were red hematemesis, SBP ≤100 mm Hg, heart rate ≥100 bpm, severe Forrest endoscopic classification, and age. Age below or over 60 years was considered as a risk factor between high-risk and low-risk NVUGIB.^[17]^ However, in our retrospective study, it was observed that in the group aged over 60 years, high-risk NVUGIB was 30.88% (105/340), and it was 34.06% (78/229) in the group aged below 60 years, without any statistical significance (*P* < 0.05). Forrest classification was also compared between patients aged below and over 60 years, and no statistical significance was obtained (*P* < 0.05). In this study, it was found that there were significant statistical differences in SBP, DBP, heartbeats, HCT, PLT, albumin, BUN, red hematemesis, red stool, palpitation, cold sweat, and shock. Multivariate logistic regression analysis showed positive high-risk variables were red hematemesis, red stool, shock, volume of hematemesis, and BUN, and negative high-risk variables were Hb, PLT, and PT (Table 2).

Further ROC analysis showed that Hb level ≤75 g/L and BUN ≥8.5 mmol/L could predict the presence of endoscopic high risk-NVUGIB, in addition to red hematemesis, red stool, and shock (Fig. 1 and Table 3). However, the sensitivities of these signs for high-risk NVUGIB were poor (10.9% for shock, 14.2% for red stool, 50.8%% for red hematemesis, 47.5% for Hb ≤75 g/L), but with high specificity (97.9% for shock, 96.4% for red stool, 85.5% for red hematemesis, 76.7% for Hb ≤75 g/L). It meant that when the NVUGIB patients presented with one of these clinical presentations, the misdiagnosis rate of high-risk NVUGIB would be low, although which would cause a relatively high missed diagnosis of high-risk NVUGIB.

In order to get a better risk system to evaluate the high-risk NVUGIB patients pre-endoscopically, we assessed these simple clinical characters in different combinations of red hematemesis, Hb ≤75 g/L, and BUN≥8.5 mmol/L, shock, red stool, each of which was scored as 1 (Table 3). It showed that the optimal Youden index was 0.448 in score 4 when more than one of these 5 clinical signs were presented, with sensitivity equal to 67.5% and specificity 77.3% (*P* < 0.05).

Actually, several other score systems in diagnosing high-risk UGIB or adverse clinical outcomes of NVUGIB patients have already been applied in the clinic.^[18]^ In the study by Ogasawara et al,^[19]^ age ≥70 years, shock on admission, Hb <8.0 g/L, serum albumin <33 g/L, exposed vessels with a diameter of ≥2 mm on the bottom of ulcers, and Forrest type Ia and Ib predicted intractable endoscopic hemostasis. It was suggested by Chen et al^[14]^ that for a patient with American Society of Anesthesiologists (ASA) score 3 to 5, Hb <70 g/L, and endoscopy within 12 hours, the probability of finding high-risk NVUGIB would be 58%. In 2014, Giese et al^[20]^ concluded that in patients with UGIB subject to after-hours endoscopy, a “high-risk” Rockall score permitted an estimation of the risk of death within 30 days, but not of rebleeding. However, Chung^[21]^ suggested that the pre-endoscopy Rockall score was not useful for predicting the need for therapeutic intervention or adverse outcomes. In 2000, Blatchford et al^[22]^ identified patients at low or high risk of needing treatment to manage their upper gastrointestinal bleeding with Blatchford score. In this study, Blatchford score, instead of Rockall score, was further to be compared with our simple risk score system. it was showed that compared with Blatchford score, the new score system was a little bit easier and more simple to apply in the clinic with better Youden index, sensitivity, and specificity (Table 3). This new score was just combined of Hb, BUN, and clinical presentations (red hematemesis, red stool, and shock). And, moreover, combined of only 3 clinical signs of red hematemesis, red stool, and Hb, a much more simple score system could be set with similar predictive accuracy of high-risk NVUGIB, with sensitivity equal to78.2%, specificity 62.3%, and Yuden index 0.405, when 1 sign was presented (*P* < 0.05).

In conclusion, when the NVUGIB patients presented with each of red hematemesis, shock, red stool, and Hb ≤75 g/L, emergent endoscopy was suggested. When BUN ≥8.5 mmol/L was included as a new risk score system, less missed diagnosis of high-risk NVUGIB would be obtained. This simple risk score could easily identify patients with high-risk NVUGIB of needing endoscopic hemostasis pre-endoscopically. Further study in the clinic was required.
